# High prevalence of *S*. *Stercoralis* infection among patients with Chagas disease: A retrospective case-control study

**DOI:** 10.1371/journal.pntd.0006199

**Published:** 2018-01-31

**Authors:** Pedro Puerta-Alcalde, Joan Gomez-Junyent, Ana Requena-Mendez, Maria Jesús Pinazo, Miriam José Álvarez-Martínez, Natalia Rodríguez, Joaquim Gascon, Jose Muñoz

**Affiliations:** 1 Infectious Diseases Department, Hospital Clínic-IDIBAPS, Barcelona, Spain; 2 International Health Department, ISGlobal, Barcelona Center for International Health Research, (CRESIB), Hospital Clínic-Universitat de Barcelona, Spain; 3 Microbiology Department, Centre Diagnòstic Biomèdic. Hospital Clínic, Barcelona, Spain; University of Washington Department of Global Health, UNITED STATES

## Abstract

**Background:**

We evaluate the association between *Trypanosoma cruzi* infection and strongyloidiasis in a cohort of Latin American (LA) migrants screened for both infections in a non-endemic setting.

**Methodology:**

Case-control study including LA individuals who were systematically screened for *T*. *cruzi* infection and strongyloidiasis between January 2013 and April 2015. Individuals were included as cases if they had a positive serological result for *Strongyloides stercoralis*. Controls were randomly selected from the cohort of individuals screened for *T*. *cruzi* infection that tested negative for *S*. *stercoralis* serology. The association between *T*. *cruzi* infection and strongyloidiasis was evaluated by logistic regression models.

**Principal findings:**

During the study period, 361 individuals were screened for both infections. 52 (14.4%) individuals had a positive serological result for strongyloidiasis (cases) and 104 participants with negative results were randomly selected as controls. 76 (48.7%) indiviuals had a positive serological result for *T*. *cruzi*. Factors associated with a positive *T*. *cruzi* serology were Bolivian origin (94.7% vs 78.7%; p = 0.003), coming from a rural area (90.8% vs 68.7%; p = 0.001), having lived in an adobe house (88.2% vs 70%; p = 0.006) and a referred contact with triatomine bugs (86.7% vs 63.3%; p = 0.001). There were more patients with a positive *S*. *stercoralis* serology among those who were infected with *T*. *cruzi* (42.1% vs 25%; p = 0.023). Epidemiological variables were not associated with a positive strongyloidiasis serology. *T*. *cruzi* infection was more frequent among those with strongyloidiasis (61.5% vs 42.3%; p = 0.023). In multivariate analysis, *T*. *cruzi* infection was associated with a two-fold increase in the odds of strongyloidiasis (OR 2.23; 95% CI 1.07–4.64; p = 0.030).

**Conclusions:**

*T*. *cruzi* infection was associated with strongyloidiasis in LA migrants attending a tropical diseases unit even after adjusting for epidemiological variables. These findings should encourage physicians in non-endemic settings to implement a systematic screening for both infections in LA individuals.

## Author summary

*Trypanosoma cruzi* infection and strongyloidiasis are neglected tropical diseases, sharing a similar epidemiological burden in Latin America and producing life-long infections, leading to high morbidity and mortality. We conducted a case-control study in a non-endemic setting to evaluate a possible relationship between both infections. High prevalence of both diseases was found and importantly, *T*. *cruzi* infection was associated with a two-fold increase in the likelihood of strongyloidiasis even after adjusting for epidemiological variables. A possible explanation is that these two infections share an epidemiological burden where they are highly prevalent, but also the fact that both diseases are strongly influenced by socioeconomical factors such as soil contamination, barefoot walking or poor healthcare systems. Moreover, immune alterations produced by *S*. *stercoralis* may predispose to *T*. *cruzi* infection. As long as screening for Chagas disease in asymptomatic Latin American adults living in Europe has shown to be cost-effective and in light of the high prevalence of strongyloidiasis found in *T*. *cruzi* infected patients, a combined screening should be considered. The potential strongyloidiasis related complications and the benefits from ivermectin therapy are additional reasons to introduce systematic screening in susceptible populations.

## Introduction

In recent years, the significant increase in the number of Latin American migrants in Europe has meant the introduction of parasitic endemic infections, such as *Trypanosoma cruzi* infection and strongyloidiasis [1–3]. Both are neglected tropical diseases (NTD), sharing a similar epidemiological profile in Latin America (LA) and producing life-long infections, usually silent, leading to high morbidity and mortality [4–6].

Chagas disease is a zoonosis endemic to LA countries [6,7], caused by hemoflagellated protozoan *Trypanosoma cruzi*, usually after contact with faeces of blood-sucking triatomines [8]. Congenital, organ transplantation and transfusion-related transmission are other principal routes of *T*. *cruzi* infection, which have been described in non-endemic areas [9]. The acute infection is followed by an asymptomatic chronic stage during years. After 20–30 years, up to 30–40% of patients will develop the symptomatic chronic phase, with cardiac and/or digestive involvement [8,10,11]. Chagas disease diagnosis in the chronic phase is based on serological tests [10,11].

Strongyloidiasis is a highly prevalent (over 30–100 million people worldwide) nematode infection, with a unique life-cycle where *Strongyloides stercoralis* females reproduce parthenogenetically to produce an autoinfective cycle that can lead to life-long and barely symptomatic carriage [5,12,13]. Nonetheless, in the context of immunosuppression, it can cause severe forms with larvae dissemination to extraintestinal organs and high mortality rates [14,15]. The diagnosis of strongyloidiasis is challenging due to irregular larvae output resulting in low sensitivity of common parasitological methods. Serology is a very sensitive test (88–95%) and it may be useful in the follow-up, as titers usually decrease after successful treatment [16–18].

In the context of migration and the increasing use of immunosuppressive treatments (steroids, monoclonal antibodies…), *T*. *cruzi* infection and strongyloidiasis have emerged as an important public health problem in Europe, North America and other areas hosting Latin American population [3,19]. Some European countries including Spain, have implemented national programmes to control transfusional and mother-to-child transmission of *T*. *cruzi* [20,21], and recent recommendations for the screening and management of strongyloidiasis in non-endemic areas have been published [22]. However, European countries are far from achieving an adequate control of the morbidity caused by these two silent chronic infections. Besides, little is known about the association between both infections in LA migrants and whether this eventual association should prompt a joint screening strategy in tropical diseases clinics.

The main aim of the present study is to evaluate the association between *T*. *cruzi* infection and strongyloidiasis in a cohort of LA migrants screened for both infections in a non-endemic setting.

## Methods

### Study setting and design

This is a retrospective case-control study performed at the Tropical Medicine and International Health Department in Hospital Clínic, Barcelona. Hospital Clínic is a tertiary teaching hospital and a national reference centre in Spain for Tropical Imported Diseases. Systematic screening of *T*. *cruzi* infection (among others) is performed among adult individuals who have lived for more than a year in endemic countries, or who are born from mothers with LA origin. Similarly, strongyloidiasis testing was incorporated in the systematic screening of these patients in January 2013. Individuals are commonly sent to our outpatient clinic referred by friends or relatives, primary healthcare professionals or they come by their own initiative.

### Participants

Eligible participants of the study were selected from the cohort of adult individuals screened for *T*. *cruzi* infection and strongyloidiasis between January 2013 and April 2015. Individuals were included as cases if they had a positive serology for *S*. *stercoralis* in the screening blood test. Individuals who had been diagnosed with strongyloidiasis prior to the study period or had previously been treated with ivermectin were excluded. Controls were randomly selected from the cohort of individuals who were screened for *T*. *cruzi* infection and tested negative for *S*. *stercoralis* serology. Two controls were randomly selected for each case included in the study.

### Study procedures

Eligible participants were invited to participate in the outpatient clinic, after signing the informed consent form. Individuals were asked for clinical and epidemiological data, which was filled in a standardized questionnaire. This contained data on sociodemographic variables, area of origin, residence in rural areas or potential risks of Chagas disease transmission (contact with *T*. *cruzi* vector, history of maternal Chagas disease or blood transfusions). Blood samples were taken to perform serological tests for *T*. *cruzi*, *S*. *stercoralis* and HIV infection. Routine hematology and biochemistry tests (including liver and renal function) were performed in all cases. Eosinophilia was defined as >500 eosinophils/mm^3^ or a percentage ≥ 5%.

Laboratory diagnosis of *T*. *cruzi* infection was established by two serological ELISA tests, following international recommendations [23]. One was a commercial ELISA with recombinant antigens (BioELISA Chagas, Biokit S.A., Barcelona, Spain), and the other was an in-house ELISA with whole *T*. *cruzi* epimastigotes antigen. Diagnosis of *T*. *cruzi* infection was defined by positivity in the two serological tests.

*S*. *stercoralis* serological screening was performed with the commercial test IVD-ELISA (IVD Research, Carlsbad, CA) which detects IgG antibodies by using somatic antigens from larvae of the parasite. A cut-off of the sample absorbance/0.2 (*i*)>1.1 is defined as positive. Individuals were also asked to provide three stool samples from different days for direct microscopic examination. Agar plate culture was also performed in at least one stool sample (when available) per individual.

### Statistical analysis

Stata version 13.1 (Stata Corporation, College Station, TX, USA) was used for statistical analyses. Categorical variables were described by counts and percentages, whereas continuous variables were expressed as means and standard deviations (SD) or medians and interquartile ranges (IQRs). The chi-square Pearson test was used to compare the distribution of categorical variables. The Mann-Whitney U test or the t-student test were used to compare the distribution of continuous variables.

To analyze the association between exposure variables and strongyloidiasis, logistic regression models were built to estimate unadjusted or adjusted odds ratios (ORs) with their 95% confidence interval (95% CI). Results were considered statistically significant if the two-tailed p-value was <0.05. The likelihood ratio test was used to obtain p-values.

### Ethical considerations

The Ethics Committee of Hospital Clínic approved this study. Data collection forms were completely anonymous. Written informed consent was obtained from participants for collecting clinical and epidemiological data.

## Results

### Cohort characteristics

**Fig 1:**
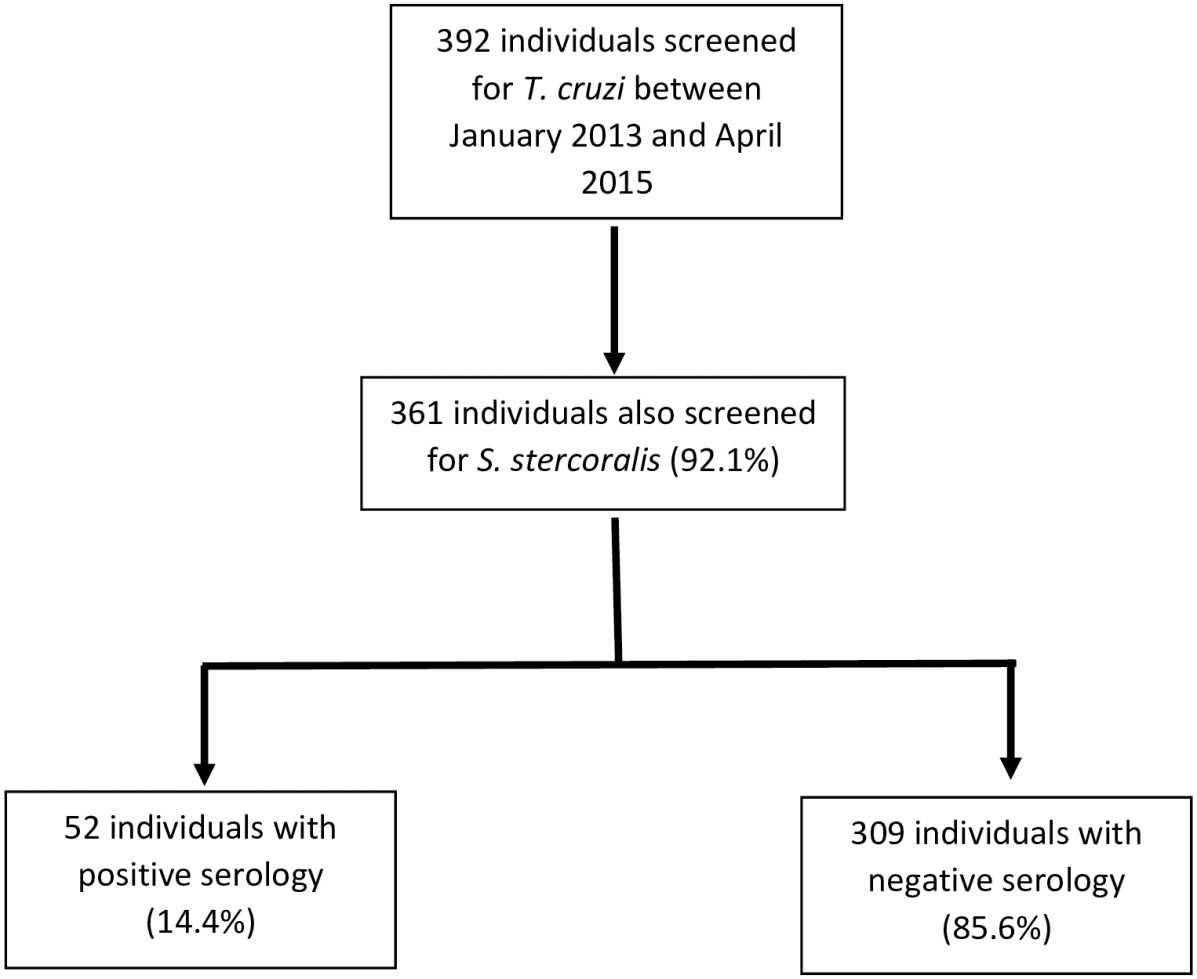
[Flowchart] Flowchart of individuals screened for *S*. *stercoralis* and finally included in the study.

During the study period, 392 patients were screened for *T*. *cruzi* infection in our center. Of these patients, 361 (92.1%) were also screened for strongyloidiasis, and were then eligible for the study (see Fig 1). Overall, 52 (14.4%) patients had a positive *S*. *stercoralis* serological result and were then included as cases and 104 out of 309 participants with negative results were randomly selected as controls by simple randomization.

Table 1 shows the baseline characteristics of the cohort. The median age of the patients was 36 years (IQR 29–43) and 100 (64.1%) were women. The vast majority came from Bolivia (135 patients, 86.5%), and the other patients came from several different LA countries. Mean time in Spain prior to screening was 8.46 years (SD 3.64). There were 124 (79.5%) patients who came from rural areas, 123 (78.8%) had lived in an adobe house and 115 (74.7%) referred having had contact with triatomine bugs. Most patients were asymptomatic and the most common complaints were abdominal bloating (19.9%), heartburn (11.5%) and abdominal pain (9%). Absolute and relative eosinophilia were present in 30 (19.2%) and 50 patients (32.1%), respectively.

**Table 1:** Baseline characteristics of 156 individuals included in the study.

Variable	n	%
**Sociodemographic characteristics**		
**Sex**		
**Women**	100	64.1%
**Men**	56	35.9%
**Age**		
**Mean years (SD)**	36.84 (11.84)	-
**< 35 years**	66	42.3%
**≥ 35 years**	90	57.7%
**Country of origin**		
**Bolivia**	135	86.5%
**Peru**	6	3.9%
**Argentina**	4	2.6%
**El Salvador**	3	1.9%
**Colombia**	2	1.3%
**Ecuador**	2	1.3%
**Paraguay**	2	1.3%
**Brazil**	1	0.6%
**Guatemala**	1	0.6%
**Years (SD) living in Spain**	8.46 (3.64)	-
**Rural area**	124	79.5%
**Adobe house**	123	78.8%
**Triatomine bug contact**	115	74.7%
**Clinical signs and diagnostic tests**		
**Clinical symptoms**		
**Abdominal pain**	14	9%
**Heartburn**	18	11.5%
**Abdominal bloating**	31	19.9%
**Skin lesions**	1	0.6%
**Eosinophil count**		
**Mean (SD)**	293 (358)	-
**Eosinophilia > 500**	30	19.2%
**Eosinophilia ≥ 1000**	7	4.5%
**Relative eosinophil count**		
**Mean % (SD)**	4.48 (4.90)	-
**Relative eosinophilia**^a^	50	32.1%
***S*. *stercoralis* positive stool samples**	8	7%
**Serologic tests**		
**Positive *T*. *cruzi* serology**	76	48.7%
***S*. *stercoralis* serology titers**^b^		
**Mean (SD)**	5.3 (4.5)	-
**< 2.50**	24	46.1%
**≥ 2.50**	28	53.8%

^a^ Defined as >5% of total leukocyte count

^b^ Of the 52 positive S. stercoralis cases

There were 76 (48.7%) patients with a positive *T*. *cruzi* serology. Most were women (52; 68.4%) and the median age was 37 years (IQR 30–43). Among those with a positive *S*. *stercoralis* serology, there were 34 (65.4%) women and the median age was 38 years (IQR 31–44). Mean serology titers were 5.3 (IQR 1.9–8.7), and 28 patients (53.8%) had titers greater than 2.50. None were positive for HIV. From the 114 (73.1%) patients who had provided at least one stool sample for examination, 2 (1.8%) had one positive sample for *S*. *stercoralis* and 6 (5.3%) had two positive samples. No other helminths were isolated from stool samples, although 25 (16%) patients had other microorganisms isolated from stool: 3 *Giardia lamblia*, 10 *Blastocystis hominis* and 12 *Entamoeba* sp.

### Evaluation of risk factors for *T*. *cruzi* infection and *S*. *stercoralis* infection

Table 2 shows the main characteristics of patients regarding to whether they had *T*. *cruzi* infection and/or strongyloidiasis.

**Table 2:** Baseline characteristics of 156 individuals included in the study, according to results on T. cruzi and strongyloidiasis serology.

Variable	*T*. *cruzi* positive (n = 76)	*T*. *cruzi* negative (n = 80)	p value	*S*. *stercoralis* positive (n = 52)	*S*. *stercoralis* negative (n = 104)	p value
**Women**	52 (68.4%)	48 (60%)	0.273	34 (65.4%)	66 (63.5%)	0.813
**Age ≥ 35 years**	47 (61.8%)	43 (53.7%)	0.307	33 (63.5%)	57 (54.8%)	0.302
**Bolivian origin**^a^	72 (94.7%)	63 (78.8%)	0.003	46 (88.5%)	89 (85.6%)	0.619
**Rural area**	69 (90.8%)	55 (68.7%)	0.001	42 (80.8%)	82 (78.8%)	0.779
**Adobe house**	67 (88.2%)	56 (70%)	0.006	41 (78.8%)	82 (78.8%)	1.0
**Triatomine bug contact**	65 (86.7%)	50 (63.3%)	0.001	40 (76.9%)	75 (73.5%)	0.647
**Absolute eosinophilia**^b^	19 (25%)	11 (13.7%)	0.075	20 (38.5%)	10 (9.62%)	< 0.001
**Relative eosinophilia**^c^	30 (39.5%)	20 (25%)	0.053	28 (53.8%)	22 (21.1%)	< 0.001
**Clinical symptoms**						
**Abdominal pain**	4 (5.3%)	10 (12.5%)	0.114	7 (13.5%)	7 (6.7%)	0.166
**Heartburn**	8 (10.5%)	10 (12.5%)	0.700	8 (15.4%)	10 (9.6%)	0.288
**Abdominal bloating**	13 (17.1%)	18 (22.5%)	0.399	12 (23.1%)	19 (18.3%)	0.478
**Positive *T*.*cruzi* serology**	-	-	-	32 (61.5%)	44 (42.3%)	0.023
**Positive *S*. *stercoralis* serology**	32 (42.1%)	20 (25%)	0.023	-	-	-
***S*. *stercoralis* serology titers > 2.50**	19 (59.4%)	9 (45%)	0.312	-	-	-

^a^ Total = 135

^b^ Defined as >500 eosinophils/mm^3^

^c^ Defined as >5% of total leukocyte count

Gender was not found to be associated with T. cruzi infection (p = 0.273) nor strongyloidiasis (p = 0.813). The proportion of patients aged 35 or older was also similar among *T*. *cruzi* (p = 0.307) and *S*. *stercoralis* (p = 0.302) infected and non-infected participants.

Factors associated with a positive *T*. *cruzi* serology were Bolivian origin (94.7% vs 78.7%; p = 0.003), coming from a rural area (90.8% vs 68.7%; p = 0.001), having lived in an adobe house (88.2% vs 70%; p = 0.006) and a referred contact with triatomine bugs (86.7% vs 63.3%; p = 0.001). There were more patients with a positive *S*. *stercoralis* serology among those who were infected with *T*. *cruzi* (42.1% vs 25%; p = 0.023).

Epidemiological variables, such as Bolivian origin (88.5% vs 85.6%; p = 0.619), coming from a rural area (80.8% vs 78.8%; p = 0.779), having lived in an adobe house (78.8% in both groups) and a referred contact with triatomine bugs (76.9% vs 73.5%; p = 0.647) were not associated with a positive strongyloidiasis serology.

*T*. *cruzi* infection was more frequent among those with strongyloidiasis (61.5% vs 42.3%; p = 0.023). No differences between both groups were found in clinical symptoms, such as abdominal pain (13.5% vs 6.7%; p = 0.166), heartburn (15.4% vs 9.6%; p = 0.288), and abdominal bloating (23.1% vs 18.3%; p = 0.478). Isolation of other microorganisms in stool samples was also not associated with strongyloidiasis (16.7% vs 25%; p = 0.300).

### Association between Chagas disease and strongyloidiasis

After adjusting for sex, age, country of origin and rural area, *T*. *cruzi* infection was associated with a two-fold increase in the odds of strongyloidiasis (OR 2.23; 95% CI 1.07–4.64; p = 0.030) (Table 3). We decided not to adjust for relative eosinophilia as this factor could cause collinearity with strongyloidiasis. Similarly, triatomine bug contact and living in an adobe house were also not included, since these variables could cause collinearity with rural area.

**Table 3:** Unadjusted and adjusted odds ratios (OR) of the association between baseline characteristics and strongyloidiasis.

Characteristic	Unadjusted OR (95% CI)	p value	Adjusted OR (95% CI)	p value
**Male sex**	0.92 (0.46–1.85)	0.813	1.02 (0.50–2.10)	0.952
**Age ≥35 years**	1.43 (0.72–2.84)	0.301	1.38 (0.68–2.79)	0.365
**Bolivian origin**	1.29 (0.47–3.55)	0.615	1.03 (0.35–3.00)	0.954
**Rural area**	1.13 (0.49–2.60)	0.778	0.81 (0.33–2.00)	0.653
***T*. *cruzi* infection**	2.18 (1.10–4.31)	0.023	2.23 (1.07–4.64)	0.030

## Discussion

In this retrospective case-control study, we offer an evaluation of the clinical and epidemiological characteristics of LA migrants screened for both *T*. *cruzi* infection and strongyloidiasis in a reference unit for tropical diseases. The most important finding of our study is the association found between both strongyloidiasis and *T*. *cruzi* infection.

Almost all individuals screened were young, with no comorbidities—probably reflecting the overall epidemiological characteristics of global population migrating for work.

The proportion of *T*. *cruzi* infection was found to be high, as in other series of imported diseases centers and known to be altered by a positive selection bias [24,25]. This figure is highly conditioned by the fact that the majority of patients came from Bolivia, which is known to be a highly endemic country for Chagas disease [6]. In addition, most of them had lived in rural areas where the high prevalence of the vector is associated to suitable conditions for transmission such as the presence of adobe houses. Expectedly, *T*. *cruzi* infection was associated with Bolivian origin, having lived in an adobe house and a referred contact with triatomine bugs.

For initially screened patients, strongyloidiasis prevalence was 14.4%. This prevalence rate seems accordant to that found in similar migrant populations in non-endemic areas [26,27], although these studies were mostly conducted in HIV patients. Nonetheless, global prevalence of strongyloidiasis is generally underestimated and data on Bolivian prevalence of this nematode infection is especially scarce [5,28,29]. Considering the potential negative impact on patients of this life-long infection [15], this high prevalence should prompt the inclusion of active screening strategies among susceptible populations from LA [22].

In our study, strongyloidiasis was neither associated to the epidemiological nor to the clinical variables recorded. It could have been expected to find an association between a positive serology and a rural origin or having lived in an adobe house [30,31]. A possible explanation for this is that the very high prevalence of these risk factors in the whole cohort (around 80%) could have masked a possible association, but it seems unlikely to be the only explanation.

We found that strongyloidiasis and *T*. *cruzi* infection were associated even after adjusting for the main epidemiological variables. Few formal studies had previously analysed the possible association between both infections [32,33]. A possible explanation is that these two infections share an epidemiological burden where they are highly prevalent, but also the fact that both diseases are strongly influenced by socioeconomical factors such as soil contamination, barefoot walking or poor healthcare systems. Moreover, Salvador et al [33] reported a co-infection rate of 18% in those already diagnosed with *T*. *cruzi* infection and, interestingly, co-infected patients were found to have a higher proportion of positive *T*. *cruzi* RT-PCR in peripheral blood. The authors suggested that strongyloidiasis induction of Th2-immune response may lead to suppression of Th1-mediated immunity and therefore it may predispose to *T*. *cruzi* infection [33,34].

A recent cost-effectiveness study has shown that screening for Chagas disease in asymptomatic Latin American adults living in Europe is a cost-effective strategy [35]. In light of the high prevalence of strongyloidiasis found in *T*. *cruzi* infected patients, and that both diseases are prevalent and silent among Latin American migrants, a combined screening should be considered. The potential strongyloidiasis related complications and the benefits from ivermectin therapy are additional reasons to introduce systematic screening in susceptible populations.

The strengths of this study are that serology was performed systematically on the first visit, minimizing a possible selection bias, and the fact that screening for both *T*. *cruzi* and strongyloidiasis was achieved in more than 90% of the patients. However, our study has some limitations that should be acknowledged. First of all, this is an observational retrospective study and had a relatively small sample size of patients with strongyloidiasis. Secondly, the diagnosis of strongyloidiasis relied solely in a positive serologic test. A limitation of serology is that it may have false-positive results due to cross-reaction with filariae and other helminthes [36], and that it does not certainly indicate current infection [16]. Though these are important issues, especially in migrant patients where multiple parasite infections are frequent [37], IVD-ELISA has shown reliable results in term of accuracy, with high positive and negative predictive values [38]. Actually, more than half of those diagnosed with strongyloidiasis had serology titers above 2.50, which was correlated with the highest positive predictive value in one study [38]. Moreover, no other helminths were isolated from stool samples and the longtime living in Spain at screening reduces the possibility of a potential cross-reaction. Lastly, another limitation of our study is that the clear predominance of Bolivian patients compels us to be cautious with the generalizability of our findings, and further studies with higher proportions of LA migrants from other countries would be necessary.

In conclusion, *T*. *cruzi* infection was found to be associated to strongyloidiasis in LA migrants attending a tropical diseases unit. These results suggest that both infections are prevalent in these individuals and increase the scarce knowledge about the possible relationship between these two parasites. Finally, our findings should encourage physicians to implement a systematic screening program for both infections in LA individuals. Further research is needed in order to explore this possible association and the underlying mechanisms.

## Supporting information

S1 Checklist STROBE Checklist. (DOCX)

## References

[1] Pérez-Ayala A, Pérez-Molina J, Norman F, Navarro M, Monge-Maillo B, Díaz-Menéndez M et al Chagas disease in Latin American migrants: a Spanish challenge. Clin Microbiol Infect. 2011;17: 1108–1113. doi: 10.1111/j.1469-0691.2010.03423.x 21073628 10.1111/j.1469-0691.2010.03423.x

[2] Requena-Méndez A, Aldasoro E, de Lazzari E, Sicuri E, Brown M, Moore DA, et al Prevalence of Chagas disease in Latin-American migrants living in Europe: a systematic review and meta-analysis. PLoS Negl Trop Dis. 2015;9: e0003540 doi: 10.1371/journal.pntd.0003540 25680190 10.1371/journal.pntd.0003540 PMC4332678

[3] Bisoffi Z, Buonfrate D, Montresor A, Requena-Méndez A, Muñoz J, Krolewiecki AJ, et al Strongyloides stercoralis: a plea for action. PLoS Negl Trop Dis. 2013;7: e2214 doi: 10.1371/journal.pntd.0002214 23675546 10.1371/journal.pntd.0002214 PMC3649953

[4] Chammartin F, Scholte RG, Guimarães LH, Tanner M, Utzinger J, Vounatsou P. Soil-transmitted helminth infection in South America: a systematic review and geostatical meta-analysis. Lancet Infect Dis. 2013;13: 507–518. doi: 10.1016/S1473-3099(13)70071-9 23562238 10.1016/S1473-3099(13)70071-9

[5] Buonfrate D, Mena MA, Angheben A, Requena-Méndez A, Muñoz J, Gobbi F, et al Prevalence of strongyloidiasis in Latin America: a systematic review of the literature. Epidemiol Infect. 2015;143: 452–460. doi: 10.1017/S0950268814001563 24990510 10.1017/S0950268814001563 PMC9507070

[6] Chagas disease in Latin America: an epidemiological update based on 2010 estimates. Wkly Epidemiol Rec. 2015;90: 33–43. 25671846

[7] Stanaway JD, Roth G. The burden of Chagas disease: estimates and challenges. Glob Heart. 2015;10: 139–144. doi: 10.1016/j.gheart.2015.06.001 26407508 10.1016/j.gheart.2015.06.001

[8] Rassi A Jr, Rassi A, Marín-Neto JA. Chagas disease. Lancet. 2010;375: 1388–1402. doi: 10.1016/S0140-6736(10)60061-X 20399979 10.1016/S0140-6736(10)60061-X

[9] Coura JR. The main sceneries of Chagas disease transmission. The vectors, blood and oral transmissions—a comprehensive review. Mem Inst Oswaldo Cruz. 2015;110: 277–282. doi: 10.1590/0074-0276140362 25466622 10.1590/0074-0276140362 PMC4489464

[10] Prata A. Clinical and epidemiological aspects of Chagas disease. Lancet Infect Dis. 2001;1: 92–100. doi: 10.1016/S1473-3099(01)00065-2 11871482 10.1016/S1473-3099(01)00065-2

[11] Bern C. Chagas’ disease. N Engl J Med. 2015;373: 456–466. doi: 10.1056/NEJMra1410150 26222561 10.1056/NEJMra1410150

[12] Genta RM. Global prevalence of strongyloidiasis: critical review with epidemiologic insights into the prevention of disseminated disease. Rev Infect Dis. 1989;11: 755–67. 2682948 10.1093/clinids/11.5.755

[13] Olsen A, van Lieshout L, Marti H, Polderman T, Polman K, Steinmann P, et al Strongyloidiasis–the most neglected of the neglected tropical diseases? Trans R Soc Trop Med Hyg. 2009;103: 967–972. doi: 10.1016/j.trstmh.2009.02.013 19328508 10.1016/j.trstmh.2009.02.013

[14] Keiser PB, Nutman TB. Strongyloides stercoralis in the immunocompromised population. Clin Microbiol Rev. 2004;17: 208–217. doi: 10.1128/CMR.17.1.208-217.2004 14726461 10.1128/CMR.17.1.208-217.2004 PMC321465

[15] Buonfrate D, Requena-Mendez A, Angheben A, Muñoz J, Gobbi F, Van Den Ende, et al Severe strongyloidiasis: a systematic review of case reports. BMC Infect Dis. 2013;13: 78 doi: 10.1186/1471-2334-13-78 23394259 10.1186/1471-2334-13-78 PMC3598958

[16] Siddiqui AA, Berk SL. Diagnosis of Strongyloides stercoralis infection. Clin Infect Dis. 2001;33: 1040–1047. doi: 10.1086/322707 11528578 10.1086/322707

[17] Requena-Mendez A, Chiodini P, Bisoffi Z, Buonfrate D, Gotuzzo E, Muñoz J. The laboratory diagnosis and follow up of strongyloidiasis: a systematic review. PLoS Negl Trop Dis. 2013;7: e2002 doi: 10.1371/journal.pntd.0002002 23350004 10.1371/journal.pntd.0002002 PMC3547839

[18] Buonfrate D, Formenti F, Perandin F, Bisoffi Z. Novel approaches to the diagnosis of Strongyloides stercoralis infection. Clin Microbiol Infect. 2015;21: 543–552. doi: 10.1016/j.cmi.2015.04.001 25887711 10.1016/j.cmi.2015.04.001

[19] Schmunis GA, Yadon ZE. Chagas disease: a Latin American health problem becoming a world health problem. Acta Trop. 2010;115: 14–21. doi: 10.1016/j.actatropica.2009.11.003 19932071 10.1016/j.actatropica.2009.11.003

[20] Requena-Méndez A, Albajar-Viñas P, Angheben A, Chiodini P, Gascón J, Muñoz J, Chagas disease COHEMI Working Group. Health policies to control Chagas disease transmission in European countries. PLoS Negl Trop Dis. 2014;8: e3245 doi: 10.1371/journal.pntd.0003245 25357193 10.1371/journal.pntd.0003245 PMC4214631

[21] Basile L, Oliveira I, Ciruela P, Plasencia A, Working Group for developing the Catalonian screening programme for congenital transmission of Chagas disease. The current screening programme for congenital transmission of Chagas disease in Catalonia, Spain. Euro Surveill. 2011;16. pii:19972. 10.2807/ese.16.38.19972-en 21958532

[22] Requena-Méndez A, Buonfrate D, Gomez-Junyent J, Zammarchi L, Bisoffi Z, Muñoz J. Evidence-Based Guidelines for Screening and Management of Strongyloidiasis in Non-Endemic Countries. Am J Trop Med Hyg. 2017 doi: 10.4269/ajtmh.16-0923 [Epub ahead of print] 28749768 10.4269/ajtmh.16-0923 PMC5590585

[23] WHO. WHO Consultation on International Biological Reference Preparations for Chagas Diagnostic Tests. WHO, Geneva, 2–3 7 2007 http://www.who.int/bloodproducts/ref_materials/WHO_Report_1st_Chagas_BRP_consultation_7-2007_final.pdf (accesed Nov 19, 2017)

[24] Salvador F, Treviño B, Sulleiro E, Pou D, Sánchez-Montalvá A, Cabezos J, et al Trypanosoma cruzi infection in a non-endemic country: Epidemiological and clinical profile. Clin Microbiol Infect. 2014;20: 706–712. doi: 10.1111/1469-0691.12443 24329884 10.1111/1469-0691.12443

[25] Meymandi SK, Forsyth CJ, Soverow J, Hernandez S, Sanchez D, Montgomery SP, et al Prevalence of Chagas Disease in the Latin American-born Population of Los Angeles. Clin Infect Dis. 2017;64: 1182–1188. doi: 10.1093/cid/cix064 28329123 10.1093/cid/cix064 PMC5399937

[26] Llenas-García J, Fiorante S, Salto E, Maseda D, Rodríguez V, Matarranz M, et al Should We Look for Strongyloides Stercoralis in Foreign-Born HIV-Infected Persons? J Immigr Minor Health. 2013;15: 796–802. doi: 10.1007/s10903-012-9756-6 23233123 10.1007/s10903-012-9756-6

[27] Ramos JM, León R, Andreu M, de Las Parras ER, Rodríguez-Díaz JC, Esteban A, et al Serological study of Trypanosoma cruzi, Strongyloides stercoralis, HIV, human T cell Lymphotropic virus (HTLV) and syphilis infections in asymptomatic Latin-American immigrants in Spain. Trans R Soc Trop Med Hyg. 2015;109: 447–453. doi: 10.1093/trstmh/trv043 26065661 10.1093/trstmh/trv043

[28] Cancrini G, Bartoloni A, Paradisi F, Nuñez LE. Parasitological observations on three Bolivian localities including rural communities, cities and institutions. Ann Trop Med Parasitol. 1989;83: 591–594. 2619373 10.1080/00034983.1989.11812392

[29] Tanner S, Leonard WR, McDade TW, Reyes-Garcia V, Godoy R, Huanca T. Influence of helminth infections on childhood nutritional status in lowland Bolivia. Am J Hum Biol. 2009;21: 651–656. doi: 10.1002/ajhb.20944 19402038 10.1002/ajhb.20944

[30] Echazú A, Bonanno D, Juarez M, Cajal SP, Heredia V, Caropresi S, et al Effect of Poor Access to Water and Sanitation As Risk Factors for Soil-Transmitted Helminth Infection: Selectiveness by the Infective Route. PLoS Negl Trop Dis. 2015;9: e0004111 doi: 10.1371/journal.pntd.0004111 26421865 10.1371/journal.pntd.0004111 PMC4589369

[31] Faria CP, Zanini GM, Dias GS, da Silva S, de Freitas MB, Almendra R, et al Geospatial distribution of intestinal parasitic infections in Rio de Janeiro (Brazil) and its association with social determinants. PLoS Negl Trop Dis. 2017;11: e0005445 doi: 10.1371/journal.pntd.0005445 28273080 10.1371/journal.pntd.0005445 PMC5358884

[32] Valerio L, Roure S, Fernández-Rivas G, Basile L, Martínez-Cuevas O, Ballesteros ÁL, et al Strongyloides stercoralis, the hidden worm. Epidemiological and clinical characteristics of 70 cases diagnosed in the North Metropolitan Area of Barcelona, Spain, 2003–2012. Trans R Soc Trop Med Hyg. 2013;107: 465–470. doi: 10.1093/trstmh/trt053 23783760 10.1093/trstmh/trt053

[33] Salvador F, Sulleiro E, Sánchez-Montalvá A, Martínez-Gallo M, Carrillo E, Molina I. Impact of Helminth Infection on the Clinical and Microbiological Presentation of Chagas Diseases in Chronically Infected Patients. PLoS Negl Trop Dis. 2016;10: e0004663 doi: 10.1371/journal.pntd.0004663 27115603 10.1371/journal.pntd.0004663 PMC4846079

[34] Eschbach ML, Klemm U, Kolbaum J, Blankenhaus B, Brattig N, Breloer M. Strongyloides ratti infection induces transient nematode-specific Th2 response and reciprocal suppression of IFN-gamma production in mice. Parasite Immunol. 2010;32: 370–383. doi: 10.1111/j.1365-3024.2010.01199.x 20500666 10.1111/j.1365-3024.2010.01199.x

[35] Requena-Méndez A, Bussion S, Aldasoro E, Jackson Y, Angheben A, Moore D, et al Cost-effectiveness of Chagas disease screening in Latin American migrants at primary health-care centres in Europe: a Markov model analysis. Lancet Glob Health. 2017;5: e439–e447. doi: 10.1016/S2214-109X(17)30073-6 28256340 10.1016/S2214-109X(17)30073-6

[36] Gam AA, Neva FA, Krotoski WA. Comparative sensitivity and specificity of ELISA and IHA for serodiagnosis of strongyloidiasis with larval antigens. Am J Trop Med Hyg. 1987;37: 157–161. 3605497 10.4269/ajtmh.1987.37.157

[37] Salas-Coronas J, Cabezas-Fernández MT, Vázquez-Villegas J, Soriano-Pérez MJ, Lozano-Serrano AB, Pérez-Camacho I, et al Evaluation of eosinophilia in immigrants in Southern Spain using tailored screening and treatment protocols: A prospective study. Travel Med Infect Dis. 2015;13: 315–321. doi: 10.1016/j.tmaid.2015.04.004 26001914 10.1016/j.tmaid.2015.04.004

[38] Bisoffi Z, Buonfrate D, Sequi M, Mejia R, Cimino RO, Krolewiecki AJ, et al Diagnostic accuracy of five serologic tests for Strongyloides stercoralis infection. PLoS Negl Trop Dis. 2014;8: e2640 doi: 10.1371/journal.pntd.0002640 24427320 10.1371/journal.pntd.0002640 PMC3890421

